# Steep Subthreshold Swing and Enhanced Illumination Stability InGaZnO Thin-Film Transistor by Plasma Oxidation on Silicon Nitride Gate Dielectric

**DOI:** 10.3390/membranes11110902

**Published:** 2021-11-22

**Authors:** Yiming Liu, Chang Liu, Houyun Qin, Chong Peng, Mingxin Lu, Zhanguo Chen, Yi Zhao

**Affiliations:** State Key Laboratory of Integrated Optoelectronics, College of Electronic Science and Engineering, Jilin University, Changchun 130012, China; yimingl20@mails.jlu.edu.cn (Y.L.); lchang16@mails.jlu.edu.cn (C.L.); qinhy18@mails.jlu.edu.cn (H.Q.); pengchong21@mails.jlu.edu.cn (C.P.); lumx19@mails.jlu.edu.cn (M.L.); czg@jlu.edu.cn (Z.C.)

**Keywords:** thin-film transistor, InGaZnO, interface traps, plasma oxidation, NBIS

## Abstract

In this paper, an InGaZnO thin-film transistor (TFT) based on plasma oxidation of silicon nitride (SiN_x_) gate dielectric with small subthreshold swing (SS) and enhanced stability under negative bias illumination stress (NBIS) have been investigated in detail. The mechanism of the high-performance InGaZnO TFT with plasma-oxidized SiN_x_ gate dielectric was also explored. The X-ray photoelectron spectroscopy (XPS) results confirmed that an oxygen-rich layer formed on the surface of the SiN_x_ layer and the amount of oxygen vacancy near the interface between SiN_x_ and InGaZnO layer was suppressed via pre-implanted oxygen on SiN_x_ gate dielectric before deposition of the InGaZnO channel layer. Moreover, the conductance method was employed to directly extract the density of the interface trap (*D_it_*) in InGaZnO TFT to verify the reduction in oxygen vacancy after plasma oxidation. The proposed InGaZnO TFT with plasma oxidation exhibited a field-effect mobility of 16.46 cm^2^/V·s, threshold voltage (*V_th_*) of −0.10 V, *I_on_*/*I_off_* over 10^8^, SS of 97 mV/decade, and *V_th_* shift of −0.37 V after NBIS. The plasma oxidation on SiN_x_ gate dielectric provides a novel approach for suppressing the interface trap for high-performance InGaZnO TFT.

## 1. Introduction

In recent decades, InGaZnO based oxide TFTs have been widely investigated to compete with conventional silicon-based TFTs for active matrix organic light-emitting display (AMOLED) due to its advantages of high field-effect mobility [1], excellent uniformity for large-scaled display panels [2], and high optical transparency in the visible spectrum [3]. Moreover, the InGaZnO shows great penitential for the application of flexible electronic devices owing to its insensitive to intrinsically distorted metal–oxygen–metal chemical bonds [4] and low-temperature fabrication process [5,6,7]. To investigate the further potential for advanced electronic applications such as high refresh rate display and low power consuming devices, the field-effect mobility, stability, and SS should be critically considered. Among the strategies of boosting the performance of InGaZnO TFTs, the modification of interface between InGaZnO and gate dielectric is one of the effective ways [8,9,10]. The plasma treatment technique has been widely applied to tailor the surface properties of semiconductors [11,12]. Additionally, the interface between the post-deposited thin film and the former layer could also be affected by plasma treatment. In this work, the plasma oxidation on SiN_x_ gate dielectric for InGaZnO TFT with fairly low SS and excellent illumination stability has been reported. The effect of plasma oxidation on the electrical characteristic, interface trap density, and chemical component for InGaZnO have been investigated in detail. The proposed plasma oxidation method on SiN_x_ gate dielectric provides a novel approach for achieving the high-performance InGaZnO TFT.

## 2. Materials and Methods

**Figure 1:**
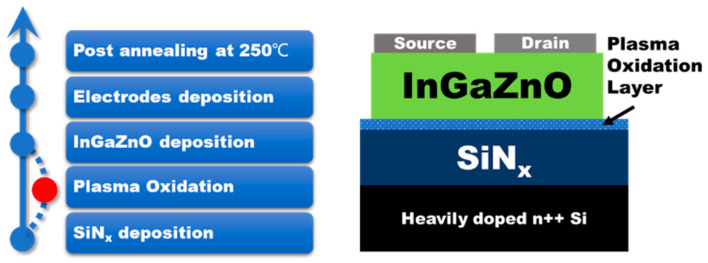
Process flow diagram and structure of InGaZnO TFT in this work.

The InGaZnO TFT was fabricated on the 210 nm-thickness SiN_x_ gate dielectric with heavily n-doped (As) Si as a gate electrode. The SiN_x_ layer was deposited by low-pressure chemical vapor deposition at the pressure of 160 mTorr from NH_3_ and SiCl_2_H_2_ precursor with the gas flow of 40 sccm and 175 sccm, respectively. The as-deposit SiN_x_ layer was treated in the oxygen plasma for 60 s. The oxygen plasma was generated by the capacity coupling configuration via a radio frequency power supply (Seren, R301) and matching box with a fixed power of 40 W under DC bias about 180 V at a constant pressure of 5 mTorr. The whole substrate was rotated at 5 revolutions per minute to keep the uniformity during the whole plasma oxidation process. Afterwards, a 30 nm thick InGaZnO channel layer was deposited by magnetron sputtering from an InGaZnO target (1:1:1 at%) with a power of 200 W in the same chamber without exposure to the atmosphere. Then, the source/drain electrodes were thermal evaporated Al metal via the shadow mask process with a channel length (*L*) of 100 μm and width (*W*) of 1000 μm, respectively. Finally, the InGaZnO TFT with plasma oxidation SiN_x_ gate dielectric (hereinafter referred to as ‘POG. TFT’) was post-annealed at 250 °C in air for 1 h. The reference sample (hereinafter referred to as ‘Ref. TFT’) was with the same fabrication sequence except for plasma oxidation on SiN_x_ dielectric. The process flow diagram and structure of InGaZnO TFTs in this work are shown in Figure 1. The electronic characteristics were evaluated using a source-meter unit (2636B, Keithley, Beaverton, OR, USA) and an LCR meter (IM3536, Hioki, Japan). The chemical status of the thin films was analyzed by XPS (Nexsa, Thermo Scientific, Waltham, MA, USA) with all the XPS data calibrated by C1s BE at 284.8 eV. Surface morphology was performed by the atomic force microscope (AFM, Dimension Icon, Bruker, Billerica, Germany).

## 3. Results

**Figure 2:**
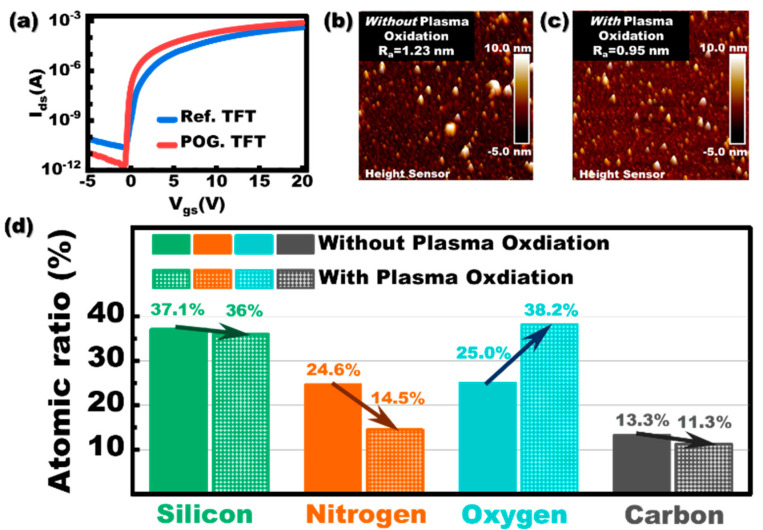
(**a**) Transfer characteristic curves for Ref. TFT and POG. TFT. The surface morphology for SiN_x_ (**b**) without plasma oxidation and (**c**) with plasma oxidation. (**d**) The atomic ratio for the surface of SiN_x_ thin film without/with plasma oxidation.

Figure 2a shows the transfer characteristics at *V_ds_* = 10 V while the *V_gs_* was swept from −5 V to 20 V with a step of 0.125 V for POG. TFT and Ref. TFT.

The field-effect mobility in saturation region is extracted from the following equation:

(1) $$\mu_{FE}=2L/WC_{ox}{\left(d\sqrt{I_{ds}}/dV_{gs}\right)}^{2}$$

and the SS is calculated by [13]:

(2) $$SS=min\left(dV_{gs}/dlogI_{ds}\right)\;$$

where *C_ox_*, *L*, *W*, *I_ds_*, and *V_gs_* are the gate capacitance per unit area, channel length, channel width, current of drain to source, and gate bias voltage, respectively. All the extracted parameters are summarized in Table 1.

**Figure 3:**
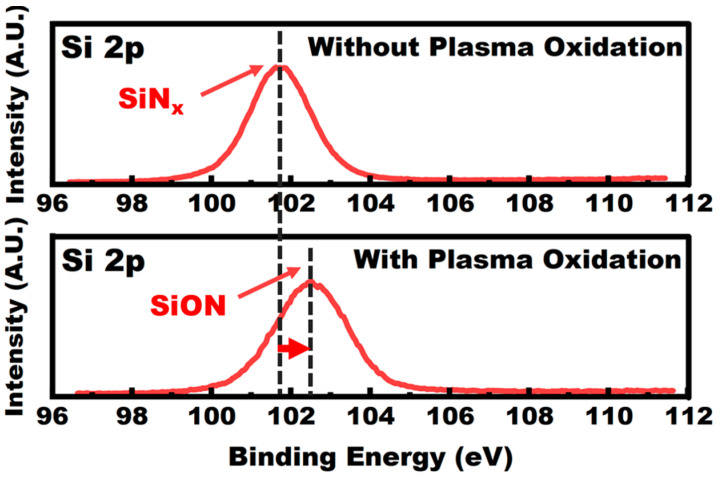
Si2p XPS results on the surface SiN_x_ film without and with plasma oxidation.

Compared with the Ref. TFT, the mobility of POG. TFT increased from 10.64 cm^2^/V·s to 16.46 cm^2^/V·s and the *V_th_* slightly shifted from 1.95 V to −0.10 V. The SS has an obvious decrease from 312 mV/decade to 97 mV/decade. Generally, the value of SS is dominated by the density of trap state in semiconductor bulk and interface trap between semiconductor and gate dielectric. The roughness of the gate dielectric could directly influence the interface between the InGaZnO and dielectric [14,15,16]. Hence, to investigate the condition of the interface, the AFM topography was obtained for the SiN_x_ sample with/without plasma oxidation under the identical process condition as described before, as shown in Figure 2b,c. The value of the surface roughness is decreased from 1.23 nm to 0.95 nm after plasma oxidation. The large cluster SiN_x_ or absorbed carbon contaminant could be partly peered off from the surface of the SiN_x_ by the plasma bombardment which provides a smoother surface for the following sputtering of InGaZnO. Different from using electron cyclotron resonance (ECR) remote plasma to treat the thin film [17], capacitive coupling was used to provide O_2_ plasma with a stronger bombardment effect to treat SiN_x_ insulators. These strongly bombarded O_2_ plasmas would treat the SiN_x_ insulators more adequately. To further verify the impact on elements composition after plasma oxidation, the elements composition by XPS for the surface of SiN_x_ thin film without/with plasma oxidation is shown in Figure 2d. For the sample of SiN_x_ without plasma oxidation, the oxygen atoms are mainly attributed to the surface absorbed oxygen from the environment on the surface of SiN_x_. After plasma oxidation, the O atoms ratio increased from 25.0% to 38.2%, the Si-O bonding would be formed on the surface of SiN_x_ thin film. To verify this speculation, the XPS spectra of the Si2p in the surface of SiN_x_ thin film were also measured. As shown in Figure 3, the Si2p binding energy on the surface of SiN_x_ at 102.5 eV (with plasma oxidation). This binding energy of Si2p is between those of the Si_3_N_4_ (101.7 eV) and SiO_2_ (103.5 eV), which indicate the formation of Si-O bonding on the surface of SiN_x_ thin film with plasma oxidation. Since the SiN_x_ thin film was mounted on the anode of the plasma generator in this work, an electric field point to the substrate could form on the SiN_x_ surface. As a result, the cations in the plasma such as O^2+^ or O^+^ could sustain surface bombardment, which causes the Si-N bonds to break and oxygen atoms could substitute partly nitrogen atoms to form an oxygen-rich layer on the SiN_x_ surface. In addition, the carbon atoms ratio has also been reduced after plasma treatment. Such carbon is mainly induced by the inevitable contamination from the vacuum chamber or transfer process. Since the carbon has been reported as an electron trap in InGaZnO [18], the reduction of carbon on the SiN_x_ surface could also increase the mobility and meliorate the SS of the InGaZnO TFT.

**Figure 4:**
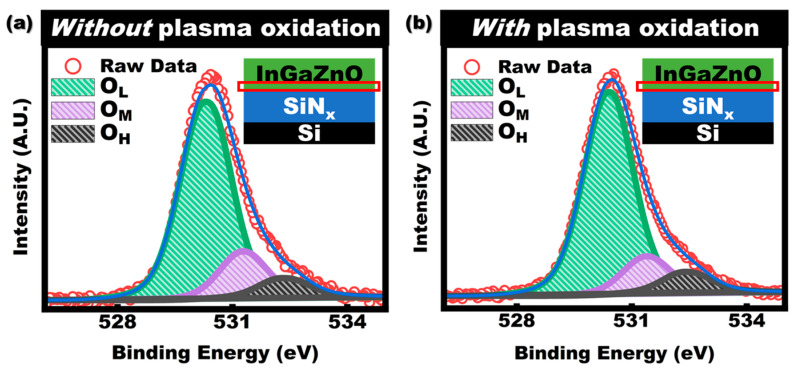
XPS results for surface of SiN_x_ thin film (**a**) without (**b**) with plasma oxidation.

Furthermore, the influence of SiN_x_ surface plasma oxidation on the post-deposit InGaZnO layer was evaluated by the O1s XPS profile at the interface between SiN_x_ and InGaZnO for the sample of with/without plasma oxidation. Figure 4a,b shows the XPS spectra of the O1s core level in InGaZnO near the interface between SiN_x_ and InGaZnO. The O1s peak of XPS spectra is deconvoluted into three peaks with a binding energy of about 530.3 eV, 531.3 eV, and 532.3 eV. The main peak centered at about 530 eV (O_L_) is related to the lattice oxygen. The peak centered at 531.3 eV (O_M_) and 532.3 eV (O_H_) is related to the oxygen vacancies and -OH hydroxide oxygen [19,20], respectively. The area ratio of O_L_:O_M_:O_H_ for the sample without plasma oxidation and with oxidation is about 1:0.25:0.10 and 1:0.18:0.12, respectively. This result indicates that the amount of oxygen vacancies in a-InGaZnO near the interface between SiN_x_ and InGaZnO is decreased after surface plasma oxidation of the SiN_x_ layer. This phenomenon could be explained by the following mechanism. After InGaZnO layer deposition, the thermal post-anneal process could cause the oxygen atoms near the interface to diffuse from the InGaZnO layer into the SiN_x_ layer, which is driven by the oxygen concentration gradient. Such a diffusion process could be partly restrained by reducing the concentration gradient via pre-implantation oxygen atoms at the SiN_x_ surface leading to a reduction in oxygen vacancies in InGaZnO near the interface between SiN_x_ and the InGaZnO layer. Since the oxygen vacancy is considered as the origin of the defects in InGaZnO [21,22], it could be deduced that the density of interface traps in InGaZnO TFTs after O_2_ plasma treatment should be decreased. On the other hand, the reduction in oxygen vacancy only near the interface between SiN_x_ and the InGaZnO layer would not significantly decrease the carrier concentration in the InGaZnO layer. Hence, the SS should also be decreased and the mobility should be increased, which is consistent with the decrease in the extracted value of SS and the increase in mobility from transfer curves of TFTs in Table 1.

**Figure 5:**
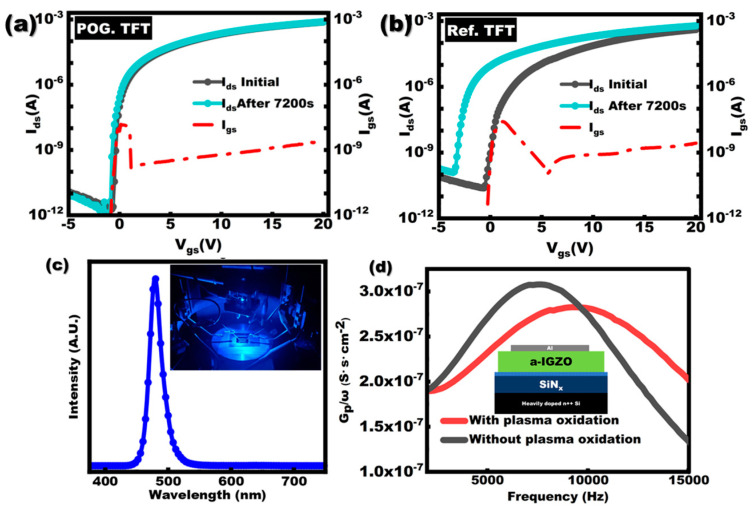
The curves of transfer characteristic before/after NBIS test for (**a**) Ref. TFT and (**b**) POG. TFT; (**c**) spectrum of blue LED used in NBIS test; (**d**) *G_p_*/*ω* as the function of frequency for Ref. and POG. MOS capacitor.

Since light illumination is inevitable during the display panel working, the NBIS test should be critically considered for InGaZnO TFTs. Furthermore, the result of NBIS could also reflect the interface status in InGaZnO TFT. Therefore, the NBIS tests were performed for both Ref. TFT and POG. TFT under a blue light-emitting diode (LED) illumination with a central wavelength of about 480 nm at full width at half maximum of 10 nm. The LED spectrum was assessed by a spectroradiometer (SpectraScan, PR-655), as shown in Figure 5c. Figure 5a,b represents the NBIS results for Ref. TFT and POG. TFT. The *V_ds_* and *V_gs_* were fixed at 0 V and −10 V, respectively. The threshold voltage shift (Δ*V_th_*) after 7200 s NBIS is −4.75 V for Ref. TFT and dramatically decreased to −0.37 V for POG. TFT. During the NBIS test, the holes and electrons are generated by the light illumination, while the oxygen vacancy could act as the hole trap to capture the photoinduced holes which are likely to drift toward the channel/dielectric interface under negative gate bias, resulting in NBIS instability in InGaZnO TFTs [23,24]. Therefore, owing to the effective decrease in the amount of oxygen vacancies near the interface between InGaZnO and SiN_x_ by plasma oxidation, the holes trapped by interfacial traps could also be reduced, resulting in a smaller Δ*V_th_* after NBIS.

To directly obtain the *D_it_* between SiN_x_ gate dielectric and the InGaZnO layer, the conductance method [25] was employed. This small-signal steady-state method has been widely used to analyze the properties of the interface trap owing to its accuracy and sensitivity in extracting the *D_it_*. The *D_it_* can be calculated from the equivalent parallel conductive (*G_p_*) divided by *ω* from the following equation:

(3) $$G_{p}/\omega =q\omega \tau_{it}D_{it}/\left[1+{\left(\omega \tau_{it}\right)}^{2}\right]\;$$

The *G_p_*/*ω* can be directly calculated from the measured equivalent parallel conductance (*G_m_*) and measured capacitance (*C_m_*) by the following express:

(4) $$G_{p}/\omega =\omega G_{m}C_{ox}^{2}/\left[G_{m}^{2}+\omega^{2}{\left(C_{ox}-C_{m}\right)}^{2}\right]\;$$

where *C_ox_* is capacitor per unit area. At maximum *Gp/ω*, the ω is equal to 1/*τit*, the *D_it_* can be expressed by the measured maximum conductance as:

(5) $$D_{it}=2/q{\left(G_{p}/\omega \right)}_{max}$$

Two metal-oxide-semiconductor (MOS) capacitors have been fabricated with a similar structure except for the plasma oxidation on the SiN_x_ surface. The *G_p_*/*ω* as a function of frequency is shown in Figure 5d. The inset is the structure of the MOS capacitor. The extracted *D_it_* is 3.02 × 10^12^ cm^−2^·eV^−1^ and 1.45 × 10^12^ cm^−2^·eV^−1^ for Ref. MOS and POG. MOS capacitors, respectively. This result also proved that the interface traps at SiN_x_/InGaZnO are reduced by the SiN_x_ surface plasma oxidation, which is consistent with the aforementioned XPS and NBIS results. Table 2 represents the performance metrics of InGaZnO TFT in this work and other reported SiN_x_-related InGaZnO TFTs. Among all SiN_x_-based InGaZnO TFTs, the TFT from our work exhibits a combination of high *I_on_*/*I_off_* and the lowest SS.

## 4. Conclusions

In this work, we demonstrated an a-InGaZnO TFT with plasma oxidation SiN_x_ gate dielectric. With plasma oxidation of SiN_x_ gate dielectric, the SS and Δ*V_th_* under NBIS were significantly improved from 312 mV/decade to 97 mV/decade and −4.75 V to −0.37 V, respectively. The plasma oxidation on SiN_x_ could provide a smoother surface and form an oxygen-rich layer at the SiN_x_/InGaZnO interface. The XPS result indicates that the amount of oxygen vacancy near the SiN_x_/InGaZnO interface was effectively reduced after plasma oxidation. Furthermore, the interface trap density has been extracted by conductance method, which shows a decrease from 3.02 × 10^12^ cm^−2^·eV^−1^ to 1.45 × 10^12^ cm^−2^·eV^−1^ after plasma oxidation. The plasma oxidation on SiN_x_ gate dielectric in this work provides a potential approach for suppressing the interface trap in SiN_x_ based InGaZnO TFT for an advanced electronic application.

## Figures and Tables

**Table 1:** Electrical parameters for InGaZnO TFT with/without SiN_x_ surface plasma oxidation.

Device	Mobility (cm^2^/V·s)	*V_th_* (V)	SS (mV/Decade)	*I_on_*/*I_off_*
Ref. TFT	10.64	1.95	312	1.68 × 10^7^
POG. TFT	16.46	−0.10	97	3.99 × 10^8^

**Table 2:** Performance metrics of InGaZnO TFT in this work and other reported SiN_x_-related InGaZnO TFTs.

Reference	Mobility (cm^2^/V·s)	*V_th_* (V)	SS (mV/decade)	*I_on_*/*I_off_*
This work	16.46	−0.10	97	3.99 × 10^8^
[26]	10.9	2.0	400	2 × 10^8^
[27]	8.79	0.81	160	3 × 10^8^
[28]	14.43	−1.25	240	−
[29]	18.1	3.4	137	1.1 × 10^8^

## Data Availability

Data are contained within the article.
